# Rehabilitation Approaches in Macular Degeneration Patients

**DOI:** 10.3389/fnsys.2016.00107

**Published:** 2016-12-27

**Authors:** Marcello Maniglia, Benoit R. Cottereau, Vincent Soler, Yves Trotter

**Affiliations:** ^1^Centre de Recherche Cerveau et Cognition, Université de Toulouse-UPSToulouse, France; ^2^Centre National de la Recherche ScientifiqueToulouse, France; ^3^Department of Psychology, University of CaliforniaRiverside, CA, USA; ^4^Department of Ophthalmology, Hopital CHU PurpanToulouse, France

**Keywords:** AMD, perceptual learning, peripheral vision, cortical plasticity, brain stimulation

## Abstract

Age related macular degeneration (AMD) is a visual disease that affects elderly population. It entails a progressive loss of central vision whose consequences are dramatic for the patient’s quality of life. Current rehabilitation programs are restricted to technical aids based on visual devices. They only temporarily improve specific visual functions such as reading skills. Considering the rapid increase of the aging population worldwide, it is crucial to intensify clinical research on AMD in order to develop simple and efficient methods that improve the patient’s visual performances in many different contexts. One very promising approach to face this challenge is based on perceptual learning (PL). Through intensive practice, PL can induce neural plasticity in sensory cortices and result in long-lasting enhancements for various perceptual tasks in both normal and visually impaired populations. A growing number of studies showed how appropriate PL protocols improve visual functions in visual disorders, namely amblyopia, presbyopia or myopia. In order to successfully apply these approaches to more severe conditions such as AMD, numerous challenges have to be overcome. Indeed, the overall elderly age of patients and the reduced cortical surface that is devoted to peripheral vision potentially limit neural plasticity in this population. In addition, ocular fixation becomes much less stable because patients have to rely on peripheral fixation spots outside the scotoma whose size keeps on evolving. The aim of this review article is to discuss the recent literature on this topic and to offer a unified approach for developing new rehabilitation programs of AMD using PL. We argue that with an appropriate experimental and training protocol that is adapted to each patient needs, PL can offer fascinating opportunities for the development of simple, non-expensive rehabilitation approaches a large spectrum of visual functions in AMD patients.

## Introduction

Age-related macular degeneration (AMD) is the leading cause of visual impairments in elderly population in western countries and affects several million of people worldwide. Starting progressively over 50 years, the end-stage AMD results in loss of central vision, usually in both eyes, with retinal scotomas extending beyond 20° of diameter (Cheung and Legge, 2005). It therefore has severe consequences for the patient autonomy and quality of life. Currently, no standard rehabilitation procedure exists (Amoaku et al., 2012). When treatments are available, they are only rarely able to reverse vision loss and are usually associated with adverse side effects (Falavarjani and Nguyen, 2013). Most of the rehabilitation programs focus on improving reading skills. These approaches generally involve vision technicians who teach the patients how to use visual aids like magnifiers, scroll text or prisms. If these devices partially help the patients to overcome their handicap, they do not improve their perceptual abilities. Moreover, training requires constant supervision, which in practice is very difficult to achieve with most of the patients.

In the recent past, new rehabilitation approaches with training based on eccentric viewing, oculomotor control and perceptual learning (PL) appeared. These approaches were quite successful at improving peripheral reading in patients (for a review see Pijnacker et al., 2011). In particular, PL seems to be very promising. This technique produces effective sensory improvement in performing perceptual tasks by training (Sagi, 2011). It can be used in clinical practice and does not require supervision of experts, nor expensive equipment. Moreover, PL mechanisms are based on long-term modifications in sensory cortex guided by neural plasticity. The aim of this review article is to describe how PL can be used to improve vision in AMD patients. In the following, we first present recent results obtained with PL in normal population or with patients suffering from central vision deficits (see the “Perceptual Learning as a Tool to Improve Visual Functions” Section), then we describe and discuss the results of very recent studies that applied PL techniques in AMD patients (see the “Perceptual Learning in AMD Patients” Section).

## Perceptual Learning as a Tool to Improve Visual Functions

### Perceptual Learning in Normal Population

The efficacy of PL has been shown in all sensory modalities, with the majority of studies focusing on visual learning. Training has been shown to improve visual perception for a variety of low-level tasks including grating detection (De Valois, 1977; Fiorentini and Berardi, 1980, 1981; Mayer, 1983), motion direction discrimination (Ball and Sekuler, 1982, 1987; Ball et al., 1983), visual search (Sireteanu and Rettenbach, 1995; Ahissar and Hochstein, 1996; Ellison and Walsh, 1998) and texture discrimination (Karni and Sagi, 1991, 1993). While the underlying neural mechanisms remain controversial (see “Perceptual Learning and Cortical Plasticity in AMD” Section), vision scientists usually agree that PL of basic visual stimulus properties induces modifications of cortical processing in early visual system, e.g., in the striate (Karni and Sagi, 1991, 1993; Saarinen and Levi, 1995; Pourtois et al., 2008) and/or extrastriate (Ahissar and Hochstein, 1996) visual cortex. In this context, the question of the specificity of PL has long been considered as an important drawback for its application in visual rehabilitation. To result in a general improvement of visual perception, the neural changes induced by PL should reach beyond early visual areas, producing modification in higher-level, non-retinotopic cortical regions (Ahissar and Hochstein, 1997; Zhang et al., 2010). Interestingly, recent studies demonstrated that specific experimental designs actually permit to transfer the improvement obtained in a low level training task (i.e., contrast detection) to higher-level visual functions (i.e., Visual Acuity (VA), Contrast Sensitivity (CS)) in both normal and visually impaired population (Levi et al., 1997; Polat et al., 2004; Tan and Fong, 2008). Other studies have also demonstrated that, under precise conditions, learning could transfer to different retinal locations. Specifically, Xiao et al. (2008) showed that with a double training paradigm, in which one retinal location is trained with a task (contrast discrimination) and another retinal position is trained with a different task (orientation discrimination), performances on the first task also increased in the second position that was not trained on this task. This result supports the involvement of higher-level visual areas that enable the transfer of learning across spatial positions. Finally, recent studies suggest that specificity of learning and amount of transfer are directly related to the difficulty of the training (Hung and Seitz, 2014). Transfer of learning seems absent for difficult trials and restored with easy trials, consistent with the reverse hierarchy theory (Ahissar and Hochstein, 1997, 2000) stating that difficult tasks induce a shrinking of the attentional window and an increase of learning specificity. Altogether, the aforementioned studies suggest that, with the appropriate experimental protocol, PL can induce general and long-lasting improvements in a number of visual abilities. For these reasons, PL has been rapidly proposed as a rehabilitation procedure in patients suffering from visual impairments.

### Perceptual Learning in Population with Central Vision Deficits

In the last 20 years, PL has been applied to the re-education of patients with central vision deficits (Levi and Polat, 1996; Levi et al., 1997). A multitude of techniques have attempted to improve vision in a variety of visual conditions (see Campana and Maniglia, 2015, for a recent research topic on practical applications of PL). A number of these studies used a low-level task based on a collinear facilitation paradigm. In this task, participants have to detect a low-contrast Gabor patch between two Gabor patches aligned collinearly (Polat and Sagi, 1993, 1994a). The flanking elements facilitate detection performances from early sensory mechanisms that are likely to involve horizontal connections in primary visual cortex (Gilbert and Wiesel, 1985, 1992; Ts’o et al., 1986; Grinvald et al., 1994; Polat and Norcia, 1996). Through PL, collinear facilitation can be improved and enhances in turn higher visual abilities that rely on inputs from earlier stages (Polat and Sagi, 1994b). This protocol has proven its efficacy in treating amblyopia, a developmental visual anomaly that reduces vision in one eye. For example, Polat et al. (2004) showed that this training improved CS and VA in the amblyopic eye, with results persisting after up to 1 year. Hussain et al. (2012) trained amblyopic patients on a flanked letter identification task and reported a reduction of critical spacing between letters in the amblyopic eye. The collinear facilitation paradigm has also been used to treat presbyopia (Polat, 2009), a common age-related visual disease that affects most of the population over 50 years and myopia (Tan and Fong, 2008). In both cases, results showed improvement of CS and VA. In conclusion, PL paradigms, based on simple training over a reduced period of time (1–2 months) can improve visual performances in a variety of visual deficits. However, all these PL paradigms target pathologies characterized by an intact retina, with the final goal of improving central vision after the emergence of optic (presbyopia and myopia) or cortical (amblyopia) abnormalities. In cases of more severe visual disease, such as a retinal damage in AMD, foveal restoration is unlikely. Therefore, PL must accomplish a different goal, that is, training a new retinal location to become the functional substitute of the fovea called Preferred Retinal Locus (PRL) and located in the peripheral retina, usually close to the scotoma. Consequently, an effective rehabilitation program for AMD must take into account the structural differences between foveal and peripheral vision.

### Perceptual Learning in Peripheral Vision

Peripheral vision is constrained by its anatomy. The cortical surface devoted to the processing of the peripheral field is indeed very small when compared to that dedicated to central vision. As a result, CS, orientation discrimination, letter acuity (Johnson et al., 1978), word identification speed (Latham and Whitaker, 1996), among others, are reduced in the periphery (Strasburger et al., 2011). Collinear facilitation, the aforementioned training configuration used for vision restoration (see the previous section), emerges at larger target-to-flanker distances in peripheral vision (Lev and Polat, 2011; Maniglia et al., 2011), in agreement with cortical magnification. Its spatial frequency tuning (Maniglia et al., 2015a) and spatial range (Maniglia et al., 2015b) are also different from those observed in foveal vision. Similarly, in the periphery, we experience visual crowding, i.e., the difficulty to identify peripheral targets when surrounded by flanking elements (Bouma, 1970). While almost absent in foveal vision, crowding represents a strong limitation in peripheral reading. Numerous PL studies aimed at improving peripheral visual abilities during crowding (Chung, 2007; Maniglia et al., 2011; Hussain et al., 2012; Chung and Truong, 2013), texture discrimination (Karni and Sagi, 1991), letter recognition (Chung et al., 2004, 2005) and reading speed (Chung et al., 2004; Yu et al., 2010a). Because VA and reading are the most impaired functions in peripheral vision of elderly people, these studies could provide a stepping-stone to develop rehabilitative protocols for AMD patients. Chung (2007) used a peripheral (10° of eccentricity) crowded letter identification task and reported an improvement in the task of 88% and a reduction of crowding extent of 38%, but no transfer to reading speed. Hussain et al. (2012) trained normal participants and amblyopic patients on crowded letter identification, with both groups showing similar improvements in the crowding reduction but no transfer to VA. Regarding reading speed, a number of studies showed that through various training protocols, such as letter identification (Chung et al., 2004; Lee et al., 2010; Yu et al., 2010a,b) word/non-word tasks (Yu et al., 2010a) and Rapid Serial Visual Presentation (RSVP; Chung et al., 2004), PL increased peripheral reading speed in healthy participants. For example, Chung et al. (2004) used a trigram letter-recognition task to increase the peripheral visual span (the number of characters that can be recognized during a fixation) that consequently improves reading speed. After four daily sessions, participants improved their peripheral reading speed by 41%. Yu et al. (2010a) compared three types of training for peripheral reading speed: RSVP, trigram letter-recognition and lexical-decision training method. RSVP is a paradigm in which words are showed one per time in rapid sequence in the same location, avoiding the implication of eye movements (Rubin and Turano, 1994). Participants in the first group improved their reading speed by 72%, the second group improved it by 54%, while participants trained with the lexical-decision improved it by 39%, which is significantly lower than the RSVP group. Bernard et al. (2012) investigated whether training on a trigram letter-recognition task can be optimized by using triplets of letters that are frequently used in the participants’ native language. However, results were not different from those obtained in the study by Chung et al. (2004). Maniglia et al. (2011) tested a collinear facilitation paradigm at intermediate eccentricities (4°), and showed that practice on this configuration also improved untrained visual functions such as peripheral CS and reduced crowding, in agreement with recent hypotheses supporting the idea that collinear facilitation and crowding might share similar neural substrates (Lev and Polat, 2011). This may have important implications for the rehabilitation of low-vision patients who must use peripheral vision to perform tasks (such as e.g., reading) that are usually processed by the fovea in normal sighted participants.

## Perceptual Learning in AMD

### Overview

So far, only a few studies investigated the application of PL in AMD patients. Previous rehabilitative approaches in AMD: (i) focused on exercises aimed at improving muscle control, eye movements and fixation (Nilsson et al., 2003), cognitive tasks (Watson et al., 1992) or used reading rehabilitation programs (RRP; Coco-Martín et al., 2013); (ii) stemmed from the optometry field rather than visual science; (iii) stressed the role of low vision therapists and the use of magnification devices; and (iv) lacked a theoretical framework of PL on neural plasticity. Chung (2011) can be considered as the first study in which a typical PL protocol (namely RSVP) was used to improve visual functions in AMD patients (see Table 1 for more details about this study and others that used PL on AMD population). Results showed an improvement in reading speed, with nonetheless an important inter-individual variability. However, no transfer of learning was observed for critical letter size, VA and fixation stability. This study had several limitations such as the absence of a control group, the identification of the PRL based on a task that differed from the one used during training (fixation vs. reading), the discrepancy between the monocular PRL profiling and the binocular training and the limited ecological value of the training task (RSVP prevents eye movements). It nonetheless demonstrated for the first time the possibility of improving residual vision in AMD through PL. Seiple et al. (2011) compared three rehabilitation protocols in AMD patients: (i) visual awareness and eccentric view training; (ii) eye movement training; and (iii) RSVP. In their sample of patients, only the eye movement training produced improvements in reading speed. This is consistent with the hypothesis that oculomotor control in patients without central vision plays a key role during reading. However, in contrast with Chung (2011), Seiple et al. (2011) did not find improvement in reading speed with RSVP. Methodological differences between the two studies can account for this discrepancy. Chung (2011) tested the patients binocularly while Seiple et al. (2011) did it monocularly, and their measures of reading speed differed (continuous text for Seiple et al., 2011; RSVP for Chung, 2011). Tarita-Nistor et al. (2014) used a paradigm similar to Chung (2011) but based on smaller words, near the reading acuity limit. The rationale was that PL is more effective when stimuli are presented around participants’ threshold, thereby inducing greater focus on the task (Tsodyks and Gilbert, 2004; Seitz and Watanabe, 2005; Sagi, 2011). Moreover, near threshold training generalizes to untrained visual functions (Polat, 2009), unlike above threshold training (Chung et al., 2012). Tarita-Nistor et al. (2014) reported improvement in reading speed (54%, similar to Chung, 2011), as well as binocular VA (from 0.54 to 0.44 LogMAR on average) and monocular VA in the better eye (but not in the worse). As reported earlier, RSVP eliminates the need for eye movements but it does represent an unusual situation outside experimental settings. Consequently, Tarita-Nistor et al. (2014) tested reading acuity and maximum reading speed with continuous text and found an improvement in both indexes (although no changes in critical print size). Finally, fixation stability improved after training in both eyes (62% in the better eye and 58% in the worse). These results highlight the importance of a PL training tailored on each patient’s thresholds, in order to maximize the effect of training and promote transfer to other visual abilities. However, the main limitations of this study are the absence of a control group (although authors used a test-retest sample of five patients, finding no changes in the measured visual abilities) and the lack of monitoring of the PRL position during training. Astle et al. (2015) trained AMD patients in a word identification task and reported a significant improvement in reading speed and baseline performances. Improvements were correlated with age with younger participants performing better than the elderly. Overall, learning curves for controls and patients were similar. The latter were however able to complete less blocks because of fatigue. In Astle et al. (2015), AMD patients were not trained in their PRL but rather at a fixed eccentricity (10°) and “asked to fixate the center of the fixation cross so that the end of the limbs appeared to extend equal distances into the peripheral visual field, even though the center of the fixation cross itself was not visible to any of the participants (i.e., it fell within the scotoma)”, a demanding task that probably explains the reduced number of trials that patients were able to complete during each session. Rosengarth et al. (2013) trained AMD patients using an oculomotor task, reporting improvements in reading speed (10 words/min) and fixation stability (30%). These improvements were however stronger between pre- and mid-test measurements than between pre- and post-training. Similarly, Plank et al. (2014) trained AMD patients with a classic Texture-Discrimination Task (TDT) in their PRL. At the end of the training, patients improved in the trained task of about 55%, with transfer of learning to Vernier Acuity. One of the reasons why little transfer is observed after TDT training might be due to the high specificity of learning for this task (Karni and Sagi, 1991). Similarly, Chung (2011) did not observe transfer of learning after training reading speed on a RSVP display, another task that might not represent an ideal transfer probe. Recently, Maniglia et al. (2016) trained AMD patients with a collinear facilitation configuration similar to the one used to treat amblyopia and myopia, a protocol that permits a better generalization of learning. Results showed that patients improved their performances not only in the trained task but also in transfer tasks such as CSF, VA and crowding reduction. Importantly, follow up tests demonstrated that this transfer of learning was retained 6 months after the end of the training.

**Table 1:** **Summary of recent visual training studies in patients with central vision loss**.

Study	Subjects	Pathology	Age	Type of training	Length of training	Improvement in the trained task	Improvement in untrained tasks (transfer of learning)
Chung (2011)	6	4 AMD, 2 Stargardt	57–82	Reading speed (RSVP)	6 sessions	+53%	No
Seiple et al. (2011)	30	Dry AMD	54–89	Experiment 1: Ecc. viewing; Experiment 2: Eye movements; Experiment 3: Reading	6 sessions (36 h)	Not reported	Reading speed: Experiment 1: −8.4 wpm Experiment 2: +27.3 wpm Experiment 3: +9.8 wpm
Coco-Martín et al. (2013)	41	Bilateral AMD	63–90	Reading Rehabilitation Program (RRP)	4 in-office sessions, 6 weeks home training	Not evaluable	**a**. Reading speed: +48.31 wpm **b**. Reading duration: +35 min **c**. Font size: +4.08 font points
Rosengarth et al. (2013)	9	AMD	55–81	Oculomotor training	12 sessions, 6 months	Not evaluable	**a**. Fixation stability: +30% **b**. Reading speed: +10 wpm
Tarita-Nistor et al. (2014)	10	Bilateral AMD	34–87	Reading speed (RSVP)	4 sessions	+54%	**a**. VA: +0.1 logMAR **b**. Fixation stability: +62% **c**. Reading speed: +53%
Plank et al. (2014)	13	8 AMD, 2 JMD, 3 CRD	47–79	Texture discrimination	6 sessions, 3 weeks	+55%	Vernier acuity: +33%
Astle et al. (2015)	5	4 dry AMD, 1 wet AMD	67–81	Word identification task	Up to 14 sessions, 30 min per session	+61%	No
Maniglia et al. (2016), Experiment 1	3	1 Stargartd, 1 AMD, 1 JMD	32–64	Contrast detection, PRL and Non-PRL	24 session, 8 weeks	+67%PRL, 38% Non-PRL	**a**. VA: 0.19 logMAR (PRL), 0.16 logMAR (Non-PRL) **b**. CSF: +25.8%
Maniglia et al. (2016), Experiment 2	4	1 CRSC, 1 macular hole, 1 CRD, 1 Best disease	49–62	Contrast detection, PRL only	19–27 sessions, 6–8 weeks	+159%	**a**. VA: +0.29 logMAR 6 months follow up: +0.15 logMAR, **b**. CSF +213% follow up: MD5: +62.8%, MD7: +325.7% **b**. Crowding: +40% follow up: +32%

*Abbreviations: AMD, Age-related macular degeneration; JMD, Juvenile macular degeneration; CRD, Cone-rod dystrophy; CRSC, Central serous chorioretinopathy; PRL, Preferrer retinal locus; Non-PRL, a symmetrical retinal locus respect to the PRL; RSVP, Rapid serial visual presentation; wpm, word per minute, CSF, Contrast sensitivity function; VA, Visual acuity, logMAR, log of the Minimum Angle of Resolution (standard measure of VA). “+” Indicates improvement in the performance, “−” Indicates worsening*.

### Perceptual Learning and Cortical Plasticity in AMD

PL aims at improving perceptual functions by promoting neural plasticity. However, it cannot always be assumed that PL-related improvements observed in normal participants can be found in AMD patients as well because of the age at which AMD usually emerges (>65 years old). Older participants might indeed show smaller training-related improvements because of reduced neural plasticity (Sunness et al., 2004; Smirnakis et al., 2005). In general, detrimental effects of age on visual abilities have been reported for several perceptual attributes. However, numerous studies showed PL-induced improvements in healthy elderly population, such as in texture discrimination (Andersen et al., 2010), motion discrimination (Ball and Sekuler, 1987; Bower and Andersen, 2012), orientation discrimination (DeLoss et al., 2014), Vernier acuity (Fahle and Daum, 1997), CS (DeLoss et al., 2015) and VA (Polat, 2009) among others. For example, Andersen et al. (2010) showed that texture discrimination significantly improved for elderly trained with near threshold stimuli, with a 3-month follow up showing retention of the improvement. Yu et al. (2010b) tested elderly population (55–76 years old) with the same task as Chung et al. (2004) and showed that perceptual improvements were smaller than those observed in younger participants. The difference in performance does not seem related to the training duration (Richards et al., 2006) but rather to the day-to-day lapse in learning effect absent in younger participants. Moreover, unlike in younger participants, learning in elderly participants was more specific and led to an improved reading speed only at the trained location and for the print size used during the training. This result is consistent with the idea that aging brain presents reduced neural plasticity. Astle et al. (2014) investigated the effect of age on PL: using a peripheral word identification task, the authors showed that older normal sighted participants improved more than younger participants and that the amount of learning was correlated with age and initial performance. The improvement in reading speed was significantly greater in the older group that reached the performance level of younger participants at the end of the training. This result is encouraging in the perspective of PL trainings for AMD since the average age of this clinical population leans on the older elderly side. These different studies show that even though PL in elderly population is far from being well understood, this method might offer interesting perspectives regarding rehabilitation strategies for patients suffering from AMD.

Nonetheless, a further concern is that we cannot assume that residual vision in AMD patients is the same as in normal population for a series of reasons: (1) the retinal lateral connections might be affected by the macular degeneration; and (2) the spontaneous cortical reorganization taking place after the lesion might produce differences in perceptual and training effects in AMD patients with respect to healthy participants. From the electrophysiological and neuroimaging perspectives, spontaneous cortical reorganization in AMD is a controversial topic: earlier electrophysiology studies agreed upon consistent evidence for cortical reorganization after retinal lesion (Darian-Smith and Gilbert, 1994; Gilbert, 1998). Using animal models such as cat and monkey, these topographic reorganizations following retinal lesions were hypothesized to arise from long-range horizontal connections formed by cortical pyramidal cells that undergo rapid and exuberant sprouting and pruning in response to removal of sensory input that can account for the topographic reorganization following retinal lesions (for review see Gilbert and Li, 2012). However, these views on spontaneous cortical reorganization were not conclusively supported by a study combining fMRI and electrophysiology in macaque (Smirnakis et al., 2005). In addition, recent neuroimaging data in human led to controversial results regarding whether and to what extent spontaneous neural plasticity exists in the lesion projection zone (LPZ), the region of the cortex formerly activated by foveal inputs. Using a large sample of patients under passive viewing, no evidence for cortical reorganization and remapping of V1 was found after retinal lesion (Baseler et al., 2011). Other studies used an active task (e.g., where patients had to detect some visual properties in the stimuli) and found significant BOLD activations in the LPZ but it is still unclear whether these activations reflected a real remapping of V1 (Baker et al., 2005; Dilks et al., 2009, 2014) or rather the unmasking of pre-existing cortico-cortical feedback inputs that lost their feed-forward input balancing from the LPZ (for a review Masuda et al., 2008; Wandell and Smirnakis, 2009). Also, smaller silent zones have been reported at the posterior pole of the occipital cortex during active compared to passive conditions possibly involving task-dependent signals from higher cortical areas Liu et al. (2010). These controversial views are also reflected in psychophysical studies. For example, Chung (2013) measured the psychophysical critical space of crowding in AMD patients, in particular the radial-tangential anisotropy, a trademark characteristic of crowding for which its spatial extension is about twice as large in the radial vs. the tangential meridian. Results showed that this anisotropy is absent in their PRL, with the shape of crowding resembling that of normal participants’ fovea rather than of periphery. Chung (2013) hypothesized that this finding might reflect a spontaneous reorganization based on the re-referencing of the oculomotor system towards the PRL rather than the fovea. Nandy and Tjan (2012) suggested that the anisotropic shape of peripheral crowding depends on saccade-confounded image statistics, since normal saccades are radial with respect to the fovea. If the PRL becomes the new reference for eye movements, the absence of radial saccades towards the fovea should reduce crowding. Consistently with this hypothesis, several studies showed a re-referencing of eye movements towards the PRL in AMD patients (White and Bedell, 1990; Whittaker et al., 1991). Finally, Chung (2013) suggested that the cortical site for spontaneous reorganization might be localized within visual areas usually associated with crowding, i.e., V1 (Nandy and Tjan, 2012), V2 (Freeman and Simoncelli, 2011), V3 (Tyler and Likova, 2007; Bi et al., 2009) or V4 (Motter, 2006). On the other hand, an article by Haun and Peli (2015) reported similar performances for AMD patients and controls in a peripheral ladder contour detection task. Since this task is probably based on the visual integration mechanisms supporting crowding (Field et al., 1993; Pelli et al., 2004; May and Hess, 2007), this result disagrees with a spontaneous cortical reorganization around the PRL.

Recently, two articles (Rosengarth et al., 2013; Plank et al., 2014) investigated PL in AMD patients and its neural correlates with fMRI. In the first study, Rosengarth et al. (2013) used an oculomotor training and reported no significant changes in BOLD signal between pre and post test in early visual (V1, V2 and V3) or higher level associative areas (LOC, fusiform gyrus, ITG). They nonetheless found positive correlation between fixation stability and BOLD signal. Similarly, Plank et al. (2014) used a TDT and showed a positive correlation between BOLD signal in early visual cortex and fixation stability at baseline as well as a positive correlation between the amount of learning and fixation stability at baseline. As reported earlier, TDT (and probably oculomotor training) might not be an ideal task to induce transfer of learning and plasticity effects, which could partially explain the lack of functional changes observed in the aforementioned studies.

### Challenges in the Use of Perceptual Learning in AMD Patients

PL in AMD patients suffers from practical and theoretical limitations such as the elderly age of patients, the lack of independence in transportation to reach clinical/lab facilities or the limited control of experimental conditions during potential at-home sessions. All these limitations concur in making clinical research with this population a challenge. It explains why one of the main goals in visual rehabilitation is to reduce the overall amount of training sessions and to develop effective at-home training paradigms. Previous studies on PL preferred to schedule training sessions over consecutive days, with the rationale that learning needs a sustained investment. However, a recent study by Chung and Truong (2013) investigating the effect of training frequency on a crowding task in normal and amblyopic participants, showed that improvement did not depend on the training frequency but rather on the number of sessions. In particular, PL literature shows that spaced practice, a training procedure that includes a break between trials (Donovan and Radosevich, 1999) produces greater improvements and longer duration, most likely because of the sleep consolidation process between sessions (Karni and Sagi, 1993). Overall, these results work in favor of applying PL in cases in which patients cannot reach the labs more than once per week.

Regarding the theoretical challenges, the application of vision models tested in healthy participants to clinical population is not always straightforward. Protocols that successfully led to performance improvements in normal participants might not be suitable for AMDs, because of overall poorer performance of AMD patients’ peripheral vision or as a consequence of partial spontaneous cortical reorganization (Chung, 2013). Another point of concern seems to be the retinal location at which the patients should be trained. The process leading to the emergence of the PRL is poorly understood and it is sill unclear why it takes longer to develop a PRL in patients than in controls with simulated central scotoma (Kwon et al., 2013). While several authors proposed the possibility of choosing a more favorable or sensitive spot (Nilsson, 1990; Nilsson et al., 2003) as a new PRL (a TRL, a trained retinal locus), a systematic monitoring is necessary, since there is no certainty that patients will retain the TRL after the end of the training. Similarly, a regular supervision of the scotoma size is a crucial step in order to control that the new PRL does not fall into the expanding scotoma. Moreover, patients might use different PRL to process different tasks and/or stimuli of different sizes. Finally, similarly to what is observed in normal participants (Hung and Seitz, 2014), the difficulty of the task seems to play a role in defining the amount of improvement that can be reached (Tarita-Nistor et al., 2014) which in addition to the duration of the sessions/blocks should therefore be adjusted for each patient’s need and possibility. Another issue with AMD research is the small number of patients tested and the common absence of a proper control group. As reported above, practical difficulties do not help recruiting a significant number of patients and samples are often rather inhomogeneous (Goodrich et al., 2004). It results in different outcomes from studies with similar protocols. Furthermore, the use of simulated central scotoma with an artificial disk in normal sighted participants as a control condition might not be the solution due to the functional difference with a physical retinal scotoma, of which the patient might not be totally aware. Actually, retinal lesions might not always induce dense scotomas, leaving insulas of residual vision that might not be well simulated by artificial scotomas (undefined borders). Moreover, the overall elderly age of AMD patients increases the risk that they also develop other health pathologies, either physical or mental. Even more, AMD patients seem in general more prone to have comorbidity, some of which can be life-threatening (Zlateva et al., 2007). This makes it challenging to induce improvement when targeting MD alone or to isolate the training contribution to MD reduction from the deleterious effects of other concomitant pathologies.

Finally, all these training protocols have the need for follow-up tests and regular post training monitoring, facing the difficulty of distinguishing the natural decay of visual abilities, due to age, disease and comorbidity, from the lack of effectiveness of the training.

## Perspectives

PL has recently become a focus of interest for its clinical applications and it stands as a promising tool to improve visual abilities in clinical population. However, as reported in the previous section, AMD patients present special needs and challenges that require the training to be as comfortable and effective as possible. New approaches to PL showed how specificity and lack of transfer can be overcome by new theoretical paradigms, such as double training (Xiao et al., 2008) or manipulation of exogenous attention (Szpiro and Carrasco, 2015). Both these two approaches induce transfer of learning from one retinal location to another and offer fascinating perspectives for more advanced forms of visual training that might improve visual abilities in different retinal spots as it would be the case in patients with multiple PRLs. Another potentially effective approach would be to focus on tasks that are known for inducing transfer of learning in peripheral vision as well (Polat et al., 2004; Polat, 2009; Maniglia et al., 2011, 2016). Finally, a more integrated PL paradigm that takes into account not only basic features or visual abilities (VA, crowding reduction, contrast sensitivity function (CSF)) but also their interactions with higher level functions during an active visual task (e.g., attention, cognitive control) to guide the re-referencing of the oculomotor system towards the PRL would probably be more beneficial on the long run.

Finally, brain stimulation, alone or coupled with PL, has been recently used to improve perceptual abilities in normal and visually impaired populations (Terney et al., 2008; Thompson et al., 2008; Olma et al., 2013; Camilleri et al., 2014b). In particular, transcranial magnetic stimulation (TMS), a technique based on the induction of a transient magnetic field that depolarizes the membrane of the stimulated neurons, has been shown to enhance CS in both normal sighted and amblyopic patients when applied over early visual areas (Thompson et al., 2008). Similarly, transcranial direct current stimulation (tDCS), a technique that consists in delivering weak (1.0–2.0 mA) electrical currents in targeted brain regions to induce modulation of cortical excitability, seems to improve motion perception (Olma et al., 2013) and line bisection (Sunwoo et al., 2013) in stroke patients and to reduce surround suppression (Spiegel et al., 2012) in normal participants. Neural modulation via brain stimulation therefore appears as a promising technique to improve perceptual function in visually impaired population, especially when considering that the neural basis of the observed improvement seems to rely on a long-lasting form of plasticity similar to long term potentiation (Stagg and Nitsche, 2011). Even more promising are results coming from recent studies in which brain stimulation is combined with a training protocol. Coupling PL with a stimulation protocol can increase visual abilities and reduce the number of training sessions necessary to observe a significant improvement, both in normal and visually impaired population (Camilleri et al., 2014a; Campana et al., 2014). As reported earlier, reducing training sessions would make PL-based programs more feasible for AMD patients with limited transportation options. Most of the trainings reported in the present review involved a large number of sessions, spanning over weeks or months, thereby causing discomfort and inducing some participants to abandon the protocol because of logistical reasons. Moreover, long trainings might be ineffective in cases of rapidly expanding degenerations. In particular, a form of non invasive transcranial electric stimulation (tES), namely transcranial Random Noise Stimulation (tRNS) may optimize PL effect by modulating synchronization of neural activity and inducing excitation (Moss et al., 2004) that in turn are associated with neuroplasticity (Grenier et al., 2001; Ponomarenko et al., 2008; Terney et al., 2008). The advantage of tRNS over tDCS is that it does not induce homeostasis in the targeted neurons because of its random frequency of stimulation (Terney et al., 2008). Few studies so far investigated the effect of tRNS on visual PL (Fertonani et al., 2011; Pirulli et al., 2013; Camilleri et al., 2014a; Campana et al., 2014). Specifically, Fertonani et al. (2011) showed how tRNS resulted in the highest amount of improvement in orientation discrimination with respect to other tES protocols. Campana et al. (2014) recently reported faster learning and greater transfer to VA and CS when PL was coupled with tRNS, both in normal sighted and visually impaired (myopia, amblyopia) population. Specifically, 2 weeks (8 sessions) of PL-tRNS combinations seem to induce the same amount of improvement in VA and CS as a 2 months (24 sessions) classic PL training (Camilleri et al., 2014a; Campana et al., 2014).

## Conclusion

**Figure 1:**
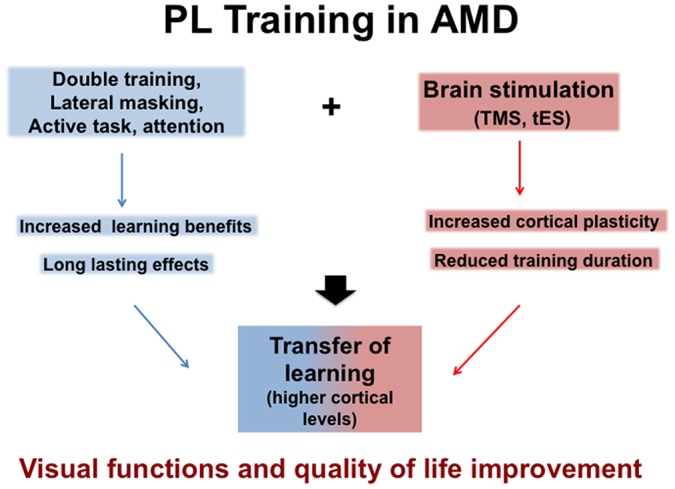
**Potential benefits of Perceptual Learning (PL) in Age Macular Degeneration (AMD) either alone or associated with brain stimulation, Transcranial Magnetic Stimulation (TMS) or transcranial Electric Stimulation (tES) that both facilitate Transfer learning (see text)**.

With the increasingly aging population, AMD is destined to become an even more common problem in western countries. Different approaches to the problem seem to focus on partial solutions or to demand a serious amount of investment both in terms of time and effort from the patient. PL seems to be a promising technique because it is easy to use (it does not require low vision therapists), affordable (it does not require expensive equipment) and comfortable (participants are trained with demanding but not uncomfortable session). Moreover PL does not just produce a temporary increase of performance, but it also promotes neural plasticity and cortical reorganization. Even more, combined approaches coupling PL with brain stimulation (electric or magnetic) promise to reduce the time needed for a significant improvement, delineating scenarios in which few weeks of training can produce long lasting changes in visual functions. They should contribute to develop efficient and appropriate rehabilitation programs to increase visual abilities and therefore quality of life of AMD patients. Figure 1 summarizes rehabilitation approaches for potential benefits.

## Author Contributions

All authors partipated in the writing of this review.

## Conflict of Interest Statement

The authors declare that the research was conducted in the absence of any commercial or financial relationships that could be construed as a potential conflict of interest.
